# Design and Test of an Integrated Measurement System for Multi-Hole Probe Calibration and Vortex Measurement

**DOI:** 10.3390/s22062376

**Published:** 2022-03-19

**Authors:** Tao Yao, Shudao Zhou, Song Ye

**Affiliations:** 1 College of Meteorology and Oceanography, National University of Defense Technology, Changsha 410073, China; yaotao19@nudt.edu.cn (T.Y.); yesong17@nudt.edu.cn (S.Y.); 2 Collaborative Innovation Center on Forecast and Evaluation of Meteorological Disasters, Nanjing University of Information Science and Technology, Nanjing 210044, China

**Keywords:** multi-hole probe, wind tunnel experiment, test system, flow around a cylinder, vortex

## Abstract

Multi-hole probes can simultaneously measure the velocity and direction of a flow field, obtain the distribution of the flow field in a three-dimensional space, and obtain the vortex information in the flow field. Moreover, a multi-hole probe needs to be calibrated while in use; therefore, a three-coordinate, multi-directional rotatable testing system, which can measure the flow field at any position and at any angle, was designed herein. A hemispherical seven-hole probe was calibrated with this test system, and the flow field around cylinders of different diameters was measured to obtain the pressure distribution and vortex shedding frequency. Furthermore, the designed test system’s ability to perform a multi-angle and multi-azimuth testing during the calibration of a multi-hole probe was verified. Simultaneously, through data mining of the multi-hole probe, vortices were measured, and periodic vortices were detected.

## 1. Introduction

In nature, the flow of fluids is not uniform, and it momentarily changes in a three-dimensional space [1], where vortices are usually present. Moreover, turbulence in the atmosphere can cause interference to aircrafts in flight and threaten the safety of lives and property. Therefore, it is necessary to monitor the vortices present in the atmosphere. Existing vortex measurement methods mostly use particle image velocimetry (PIV) technology to obtain the particle motion state within a flow field and to identify a vortex in the flow field. This is a tracer-measurement method. However, the imaging particles interfere with the flow field and are not suitable for large-scale flow-field measurements [2,3]. Vortices are essentially a state of fluid movement in a three-dimensional space [4], and if the fluid’s velocity and direction can be measured, the existence of vortices in the flow field can also be measured.

A multi-hole probe is usually used to measure the three-dimensional flow field due to its simple structure and strong environmental adaptability. By measuring the pressure of the probe hole, the velocity and three-dimensional direction of the flow field can be obtained simultaneously [5,6,7,8]. The size of the multi-hole probe is generally in millimeters, and errors inevitably occur during processing. Even if the processing error is small, the measurement results of the probe will still be affected [9]; therefore, each probe needs to be calibrated before being implemented in practice. Focusing on the calibration and measurement characteristics of a multi-hole probe, Wu et al. [10] and Georgiou and Milidonis [11] designed a test system that can control the probe’s rotation in multiple directions at a fixed position. Additionally, the test system designed by Shaw-Ward et al. [12,13] can control the up, down, forward, and backward movement of a probe, and it can also rotate the probe around its axis. However, these multi-hole probe test systems have their limitations.They only considering the application scenarios at the time can not meet the three-dimensional space omni-directional measurement.

In order to make up for the shortcomings of existing measurement systems, a three-dimensional, multi-directional rotatable measurement system, which can move in three directions in a three-dimensional space and combine pitch, yaw, and rotation around the central axis of the probe, was designed. The measurement system can perform multi-hole probe calibration in any position and in any direction, and enables simultaneous measurement of 3D velocity and vortex. A hemispherical seven-hole probe was calibrated and tested using this test system. The hemispherical seven-hole probe was used to measure the flow field around a cylinder, and the pressure distribution and vortex shedding frequency were subsequently obtained. The ability of the test system to calibrate the multi-hole probe and detect a vortex using the multi-hole probe was also verified, and this lays the foundations for future vortex measurement studies.

## 2. Design of the Test System

**Figure 1:**
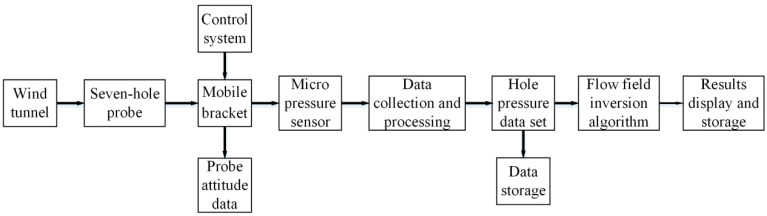
Overall design of the wind tunnel test system.

During the measurement and calibration of the multi-hole probe, fluid flowed to the probe from different directions. In the wind tunnel experiment, the flow field direction remained unchanged, the probe was adjusted to different angles of attack and roll angles, and the probe position and pressure data within the hole needed to be recorded in real time. For this purpose, an experimental test system was designed, and its composition block diagram is shown in Figure 1. The experiment environment allowed the multi-hole probe to be placed in the wind tunnel experiment cabin, and it also allowed the probe to be fixed on the mobile bracket. The PC sent instructions to the servo controller through an RS485 cable. Upon receiving the instructions, the servo controller controlled the motor to move the probe to a corresponding coordinate point, and it also simultaneously started the rotating motor to adjust the position of the probe. The pressure data were connected to the pressure sensor outside the experiment chamber through a hose, and the pressure data were collected through the data acquisition system. The acquired data and status information were then filtered, analyzed, summarized, calculated, and sorted into a data set.

### 2.1. Structural Parameters of the Hemispherical Seven-Hole Probe

**Figure 2:**
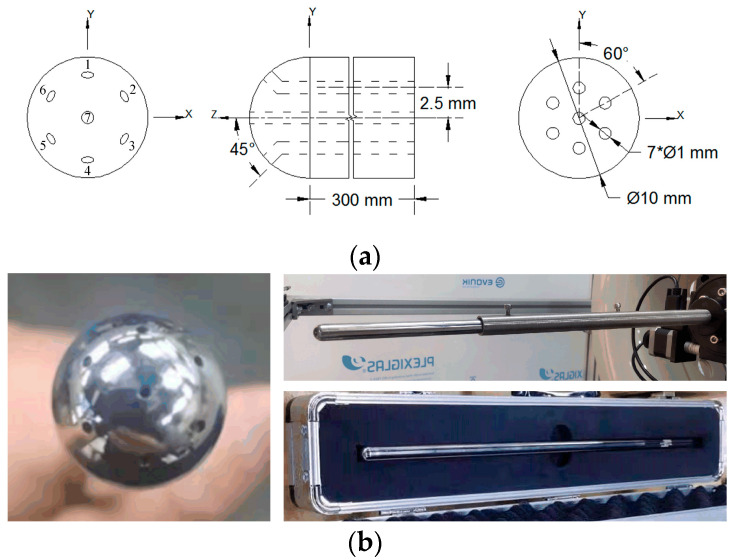
Picture of hemispherical seven-hole probe. (**a**) Schematic diagram of the probe structure, and (**b**) Photograph of the probe.

According to the conclusions from the optimization of the multi-hole probe head structure [14,15], and considering the processing accuracy, it is necessary to select a probe with a suitable diameter. A hemispherical seven-hole probe, as shown in Figure 2, was customized. The diameter of the probe and the diameter of the hemispherical head were both 10 mm, the probe length was 300 mm, the hole size was 1 mm, and the hole direction was perpendicular to the probe surface. The structural diagram is shown in Figure 2a, and a photograph of the structure is shown in Figure 2b. The probe was fixed on the mobile bracket through a sleeve, and its tail was connected to the pressure sensor through a pressure hose to obtain the pressure data from each of the seven holes.

### 2.2. Straight Open Wind Tunnel Parameters

**Figure 3:**
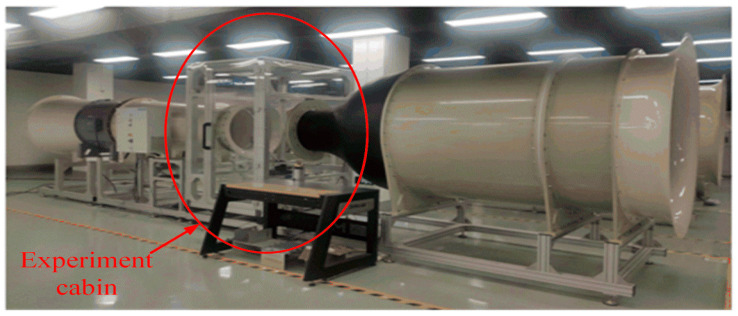
Straight open wind tunnel.

A low-speed straight open wind tunnel was used to provide the stable flow field required for the calibration and testing of the probe. The parameters of the wind tunnel are listed in Table 1, and the actual wind tunnel is illustrated in Figure 3. The cross-sectional diameter of the experimental section of the wind tunnel is 0.6 m, the length of the experimental section is 1 m, and the wind speed ranges from 0.2 to 40 m/s. The uniformity of the flow field at the exit of the wind tunnel was tested, and the turbulence was less than 0.5%; at the center point, it was less than 0.1%, and the air flow deflection angle was less than 1°. The multi-hole probe and the corresponding fixed mobile bracket were all placed inside the experimental cabin, and the air pressure, temperature, and humidity of the environment within the experimental cabin were monitored in real time using independent sensors.

### 2.3. Three-Coordinate Mobile Bracket

**Figure 4:**
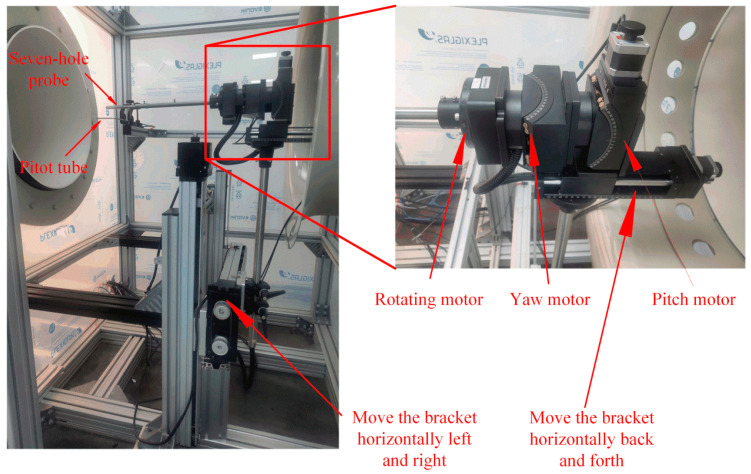
Three-coordinate mobile bracket.

Once the probe was calibrated in the wind tunnel, the probe needed to be adjusted and rotated on the pitch and yaw planes; therefore, the pressure values of the seven holes at different positions were recorded simultaneously to establish a calibration pressure dataset. Considering the diversified needs of calibration and testing, a mobile bracket with three-dimensional coordinate axis movement and independent rotation around the three axes was designed. The hemispherical seven-hole probe was fixed on the coordinate mobile bracket through a sleeve, and this structure is shown in Figure 4. The moving bracket is composed of a precision screw, a stepping motor, and a control circuit. The positional range of the moving bracket in the horizontal and vertical directions was ±300 mm, the front and back movement range was ±100 mm, and the step length was 1 mm. Through the rotating motor, the probe can be rotated by 360° around its axis with a step angle of 1°. The yaw and pitch motors control the probe’s rotation in the yaw and pitch directions, with a rotation range of ±45° and a step angle of 1°. Through the control circuit, the mobile bracket can be controlled to move to any point for measurement, and continuous movement measurements in the positional space can also be realized. During the measurement, the angle of attack, *θ*, between the incoming flow and the probe was controlled by the yaw and pitch motors, and the azimuth angle, *ϕ*, was controlled by the rotating motor so that the incoming flow reached the probe from any direction.

## 3. Multi-Hole Probe Calibration and Measurement

### 3.1. Calibration Method

The traditional multi-hole probe calibration method regards the probe as a “black box”, and the calibration is completely dependent on the wind tunnel experiment. For a 7-hole probe, there may be thousands of calibration points [16], which makes the probe calibration expensive. Therefore, we propose a new calibration method that reduces the number of calibration points required.

The multi-hole probe measured the flow field using the pore pressure in the corresponding flow when the fluid flowed around the probe head. For a sphere in the flow field, the velocity of a point on the sphere can be regarded as a function of the total angle *θ* from the stagnation point, and the velocity of any point on the sphere surface can be expressed as

(1) $$V(\theta )=\frac{3}{2}U_{\infty }\sin\theta ,$$

where $U_{\infty }$ is the incoming flow velocity. In this case, the conservation of mechanical energy is satisfied. According to the Bernoulli equation, the relationship between the pressure at any point on the sphere and the incoming flow velocity can be expressed as [17]

(2) $$p_{i}+\frac{1}{2}\rho V^{2}=p_{s}+\frac{1}{2}\rho U_{\infty }^{2},$$

where $p_{i}$ is the pressure at any point *i* on the sphere, $p_{s}$ is the static pressure of the flow field, and *V* is the velocity at any point *i* on the sphere. The dimensionless pressure coefficient $f_{i}$ of a point on the sphere can be defined as the pressure $p_{i}$ of a point on the sphere minus the static pressure $p_{s}$, and then divided by the dynamic pressure *q* as follows:

(3) $$f_{i}(\theta )=\frac{p_{i}-p_{s}}{q},$$

From the simultaneous Equations (1)–(3), the dimensionless pressure coefficient at a point on the sphere can be obtained using as

(4) $$f_{i}(\theta )=\frac{9}{4}\cos^{2}\theta -\frac{5}{4},$$

**Figure 5:**
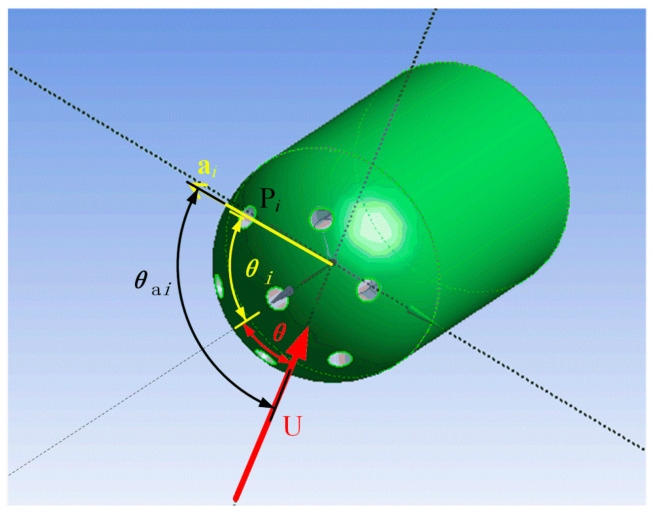
Calibration coordinate system of the hemispherical seven-hole probe.

For a hemispherical seven-hole probe, $a_{i}$ is defined as the direction vector pointing from the center of the sphere to the *i*-th hole, $a_{1}$ is the direction vector of the central hole, $\theta_{i}$ and $\phi_{i}$ are the angle of attack and azimuth of the direction vector represented by the *i*-th hole in the coordinate system, respectively, as shown in Figure 5. According to this, the hole direction and incoming flow direction are linked to the angles $\theta_{i}$ and $\phi_{i}$.

The direction vector of any *i*-th hole can be expressed as

(5) $$a_{i}=r\sin\theta_{i}i+r\sin\theta_{i}\sin\phi_{i}j+r\sin\theta_{i}\cos\phi_{i}k,$$

Similarly, the incoming flow vector can be expressed as

(6) U=Ucosθi+Usinθsinϕj+Usinθcosϕk,

where *θ* is the angle between the incoming flow and the probe axis, and *U* is the incoming flow velocity.

Then, the angle $\theta_{ai}$ between the incoming flow and the *i*-th hole can be expressed as

(7) $$\cos\theta_{ai}=\frac{U\cdot a}{\left|U\right|\left|a\right|}=\cos\theta \cos\theta_{i}+\sin\theta \sin\phi \sin\theta_{i}\sin\phi_{i}+\sin\theta \cos\phi \sin\theta_{i}\cos\phi_{i},$$

Substituting the angle relation of Equation (7) into Equation (4), we obtain

(8) $$\begin{array}{c} \frac{p_{i}-p_{s}}{\rho }=\frac{9}{8}(\cos^{2}\theta_{i}u^{2}+\sin^{2}\theta_{i}\sin^{2}\phi_{i}v^{2}+\sin^{2}\theta_{i}\cos^{2}\phi_{i}w^{2}\\ +2\cos\theta_{i}\sin\theta_{i}\sin\phi_{i}uv+2\cos\theta_{i}\sin\theta_{i}\cos\phi_{i}uw+\\ 2\sin^{2}\theta_{i}\sin\phi_{i}\cos\phi_{i}vw)-\frac{5}{8}(u^{2}+v^{2}+w^{2}) \end{array}$$,

Because the hole angle $\theta_{i}$ and azimuth angle $\phi_{i}$ of each *i*-th hole of the processed probe have now been determined, they can be replaced with different parameters, i.e.,

(9) $$\frac{p_{i}-p_{s}}{\rho }=A_{i}u^{2}+B_{i}v^{2}+C_{i}w^{2}+D_{i}uv+E_{i}uw+F_{i}vw,$$

where

(10) $$\left\{ \begin{array}{l} A_{i}=\frac{9}{8}\cos^{2}\theta_{i}-\frac{5}{8}\\ B_{i}=\frac{9}{8}\sin^{2}\theta_{i}\sin^{2}\phi_{i}-\frac{5}{8}\\ C_{i}=\frac{9}{8}\sin^{2}\theta_{i}\cos^{2}\phi_{i}-\frac{5}{8}\\ D_{i}=\frac{9}{4}\sin\theta_{i}\cos\theta_{i}\sin\phi_{i}\\ E_{i}=\frac{9}{4}\sin\theta_{i}\cos\theta_{i}\cos\phi_{i}\\ F_{i}=\frac{9}{4}\sin^{2}\theta_{i}\cos\theta_{i}\sin\phi_{i} \end{array}\right.,$$

Equation (9) is the pressure–velocity parameterized equation that can directly connect the flow field characteristics with the pressure in the hole. It is clear that when the hole angle *θ* is determined, $A_{i}-F_{i}$ are constants, but considering the machining error, the hole position cannot be completely accurate; therefore, it is necessary to re-determine the value of the parameters $A_{i}-F_{i}$ during calibration. After completing the calibration experiment to obtain the calibration data set and substituting the measured pressure and the known flow field velocity into Equation (9), the following equations can be obtained:

(11) $$\left(\begin{array}{c} {\left(\frac{p_{i}-p_{s}}{\rho }\right)}_{1}\\ {\left(\frac{p_{i}-p_{s}}{\rho }\right)}_{2}\\ \vdots \\ {\left(\frac{p_{i}-p_{s}}{\rho }\right)}_{n} \end{array}\right)=\left(\begin{array}{cccccc} u_{1}^{2} & v_{1}^{2} & w_{1}^{2} & uv_{1} & uw_{1} & vw_{1}\\ u_{2}^{2} & v_{2}^{2} & w_{2}^{2} & uv_{2} & uw_{2} & vw_{2}\\ \vdots & \vdots & \vdots & \vdots & \vdots & \vdots \\ u_{n}^{2} & v_{n}^{2} & w_{n}^{2} & uv_{n} & uw_{n} & vw_{n} \end{array}\right)\times \left(\begin{array}{c} A_{i}\\ B_{i}\\ C_{i}\\ D_{i}\\ E_{i}\\ F_{i} \end{array}\right),$$

The corresponding matrix form is

(12) $$P_{i}=X\times b_{i},$$

The parameters $A_{i}-F_{i}$ can then be obtained by the following formula

(13) $$b_{i}={\left(X^{T}X\right)}^{-1}X^{T}\times P_{i},$$

### 3.2. Calibration and Measurement Steps

**Figure 6:**
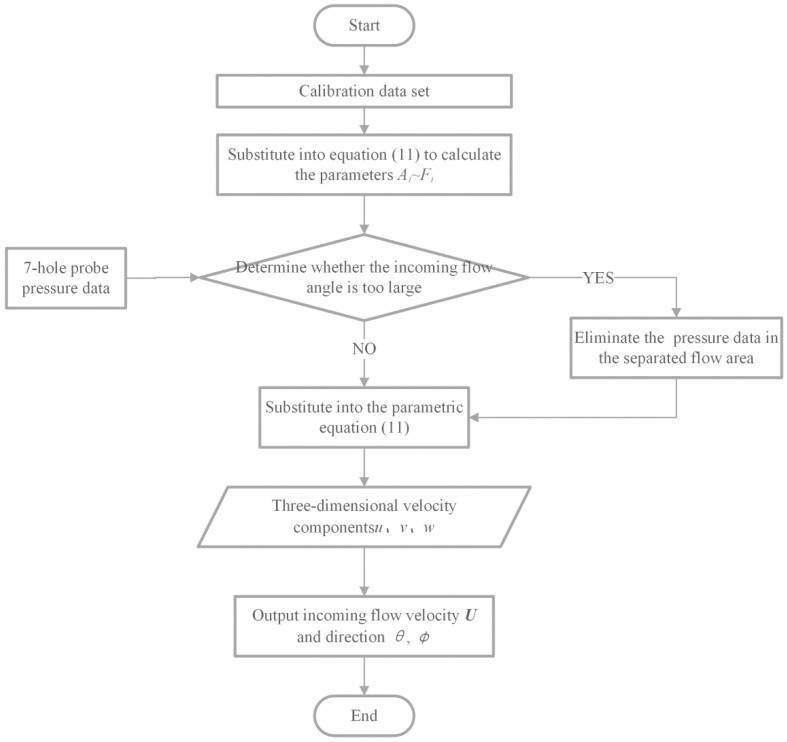
Flowchart of probe calibration and measurement.

Before the flow field measurements are carried out, the probe must be placed in a uniform flow field for calibration. After calibration, the measured pressure data in the hole are substituted into the pressure-velocity parameterization equation to obtain the flow field velocity and direction. A flow chart of the calibration and measurement is shown in Figure 6, and the processes are as follows:

1. Adjust the initial position of the probe, by changing the pitch and yaw angle to make the pressure of the outer six holes equal when the flow field is stable.
2. Change the velocity of the flow field and the angle of the probe and record the pressure of seven holes under the flow conditions of known velocity and direction to obtain a calibration data set.
3. According to the calibration pressure data set, select six groups of pressure data measured at different angles of attack and azimuth and substitute them into the calculation parameters $A_{i}-F_{i}$ using Equation (11).
4. Substitute the obtained parameter group $A_{i}-F_{i}$ back into Equation (11) to obtain the pressure-velocity parameterized equation.
5. Check whether the pressure data of seven holes are out of range during measurement.
6. Substitute the pressure data of the seven holes into Equation (11), and calculate the three-dimensional velocity components *u*, *v*, and *w* at this time.
7. Calculate the speed and direction of the incoming flow according to the angle relationship.

### 3.3. Calibration Results

The hemispherical seven-hole probe was calibrated according to the calibration and measurement steps in Figure 6. After the calibration, when the wind speed is 10, 20, 30 and 40 m/s, test points with different attack angles and roll angles are selected for testing. The measured velocity *U* and the three-dimensional velocity components *u*, *v*, and *w* are calculated according to the pressure-velocity parameterization Equation (11), and the velocity error and angle error are shown in Table 2. It can be seen from Table 2 that the maximum velocity error is approximately 6%, and the attack angle and roll angle error are approximately 5°. It shows that this calibration method can complete the calibration of the 7-hole probe well.

## 4. Vortex Measurement

### 4.1. Measurement of the Flow Field around a Cylinder

**Figure 7:**
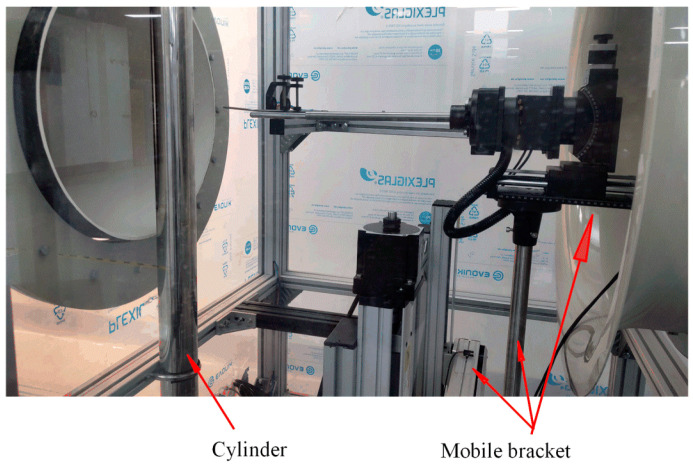
Installation position of the cylinder.

**Figure 8:**
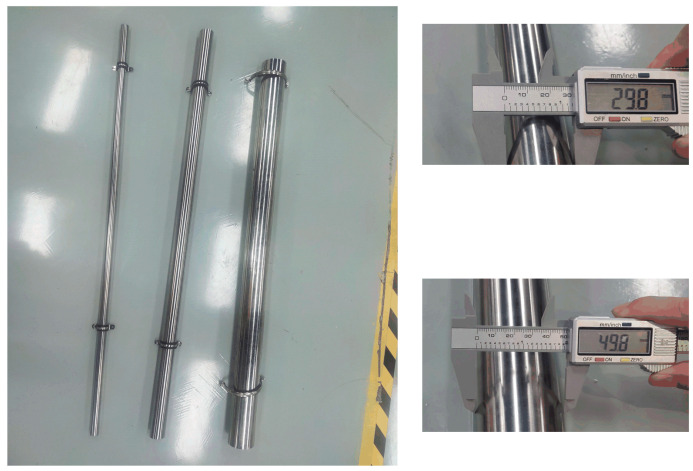
Cylinders of different sizes.

An experimental environment for the flow around a cylinder was set up to verify the test system and develop a multi-hole probe vortex measurement method. The surrounding flow column was installed and fixed near the outlet of the wind tunnel, as shown in Figure 7. The multi-hole probe was controlled to move in the three-dimensional space using the mobile bracket to realize continuous measurements at different positional measurement points. In this experiment, cylinders with diameters of 3, 5, and 9 cm were selected. Their surfaces were smoothened and the variation of the flow field within the cylinders of different diameters at different speeds were analyzed. The surrounding flow of cylinder is illustrated in Figure 8.

**Figure 9:**
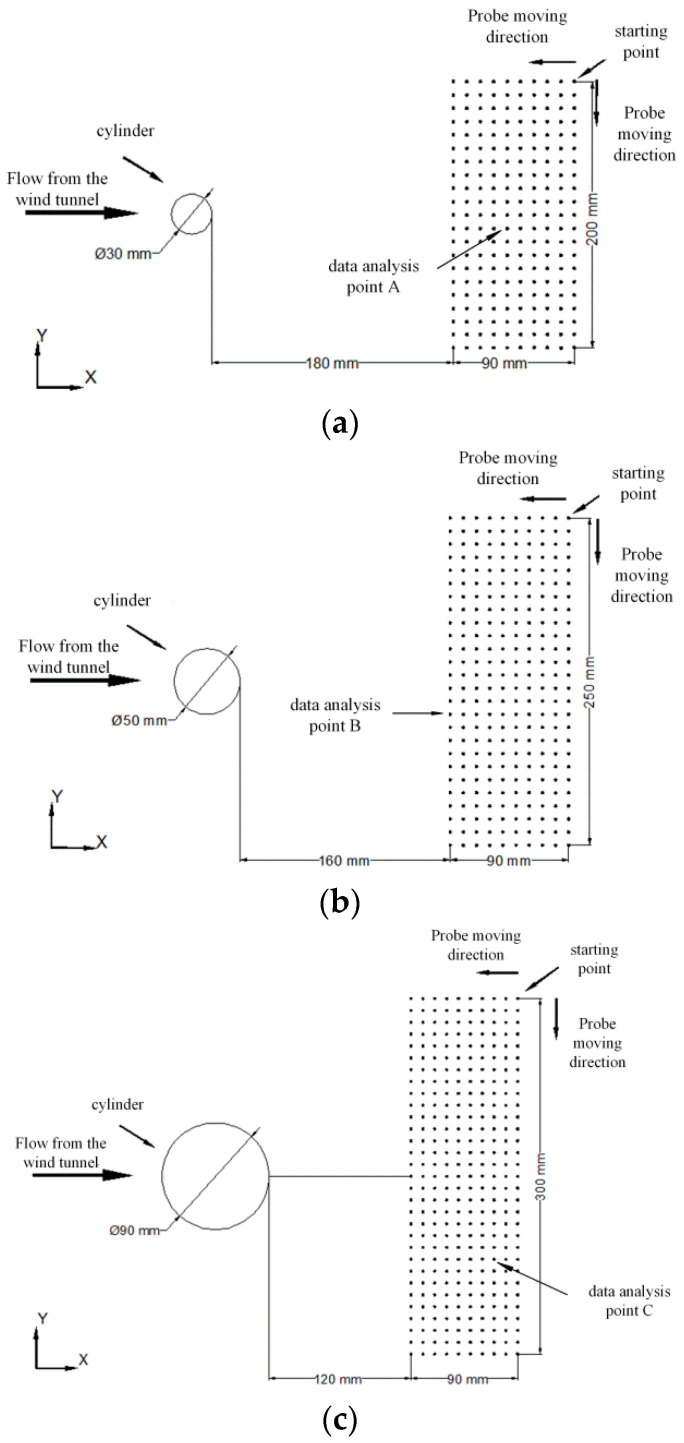
Distribution of test points for cylinders with different diameters. (**a**) 3 cm cylindrical test point. (**b**) 5 cm cylindrical test point. (**c**) 9 cm cylindrical test point.

The specific experimental steps are as follows:

1. Calibrate the multi-hole probe and adjust the initial position.
2. Divide the test points, as shown in Figure 9, for cylinders of different diameters, and move the multi-hole probe to the initial point.
3. Install a cylinder with a diameter of 3 cm and measure sequentially from the initial point in the y direction and then in the x direction. Each test point should be kept constant for 5 s after being positioned, and the pressure data of the hole are to be collected after the flow field is stable. After all points are measured, a dataset is formed.
4. Change the velocity of the flow field and set the velocity of the flow field to 5, 10, and 15 m/s, respectively. The test process is time consuming, and the environmental parameters need to be recorded at the beginning and end of the measurement to avoid long-term environmental changes that may affect the measurement.
5. Steps 3 and steps 4 are repeated with cylinders of different diameters.

### 4.2. Pressure Distribution after Flow around a Cylinder

**Figure 10:**
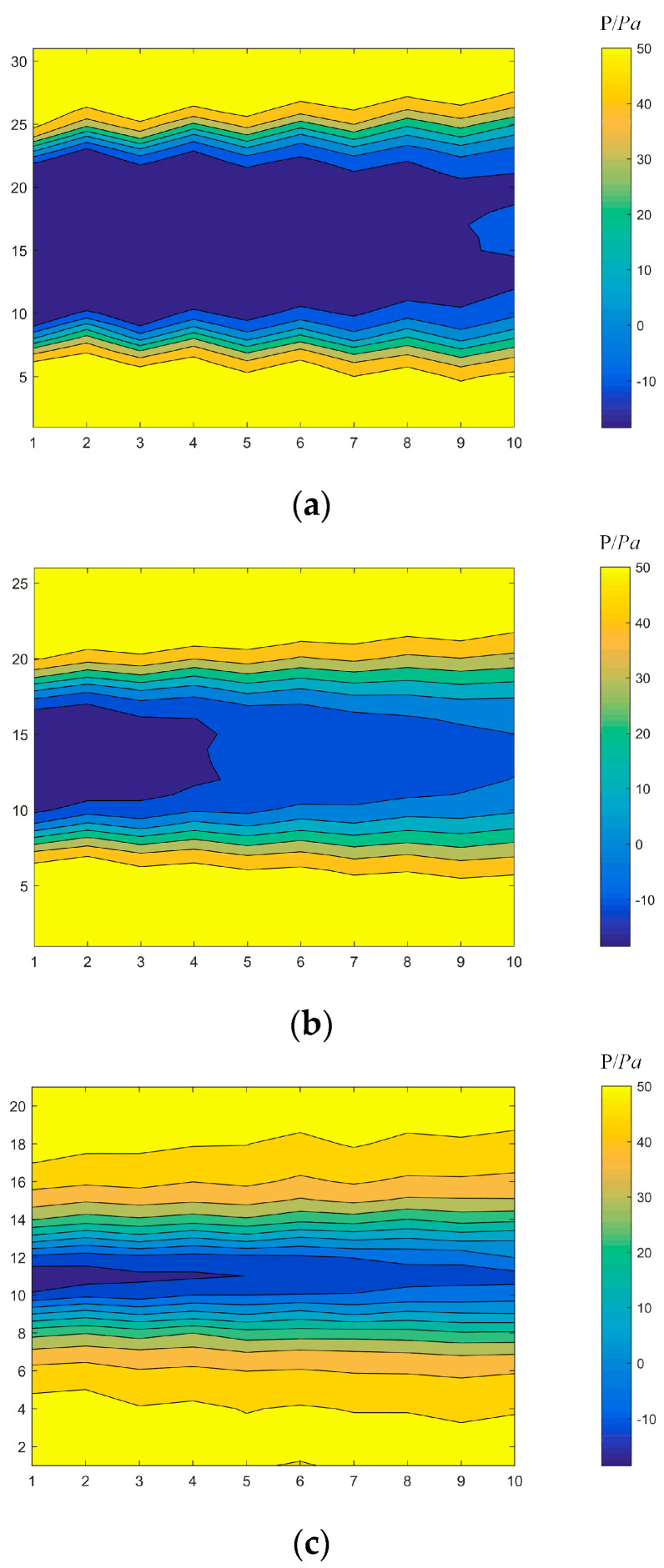
Pressure cloud diagram of flow around cylinders with diameters of (**a**) 9 cm, (**b**) 5 cm, and (**c**) 3 cm.

By analyzing the experimental test data, the flow field around different cylinders was measured, and the resulting pressure cloud diagrams of the flow fields are shown in Figure 10. Due to the limited space within the experimental cabin, for the 9 cm diameter cylindrical measuring point distribution, the near-field pressure distribution was obtained at a position closer to the cylinder. For the measurement point distribution of the cylinder with a diameter of 3 cm, the pressure distribution at the far end was obtained at a distance of six times the diameter of the cylinder. It can be seen from Figure 10 that approximately all the 9 cm cylindrical measuring points were in the low-pressure area, and the length of the 5 cm cylindrical low-pressure area was only 1/3 of the test area. The low-pressure area of the 3 cm cylindrical measuring point became more reduced, and at the end of the wake of the flow around the cylinder, the flow field was gradually stabilized. The pressure distribution of the flow field around all three cylinders of different diameters was consistent with the theoretical results. There was a wide low-pressure zone closer to the cylinder, and as the distance was increased, the low-pressure zone gradually decreased and transitioned into a stable flow field.

### 4.3. Analysis of the Frequency of Vortex Shedding in the Flow Field around a Cylinder

**Figure 11:**
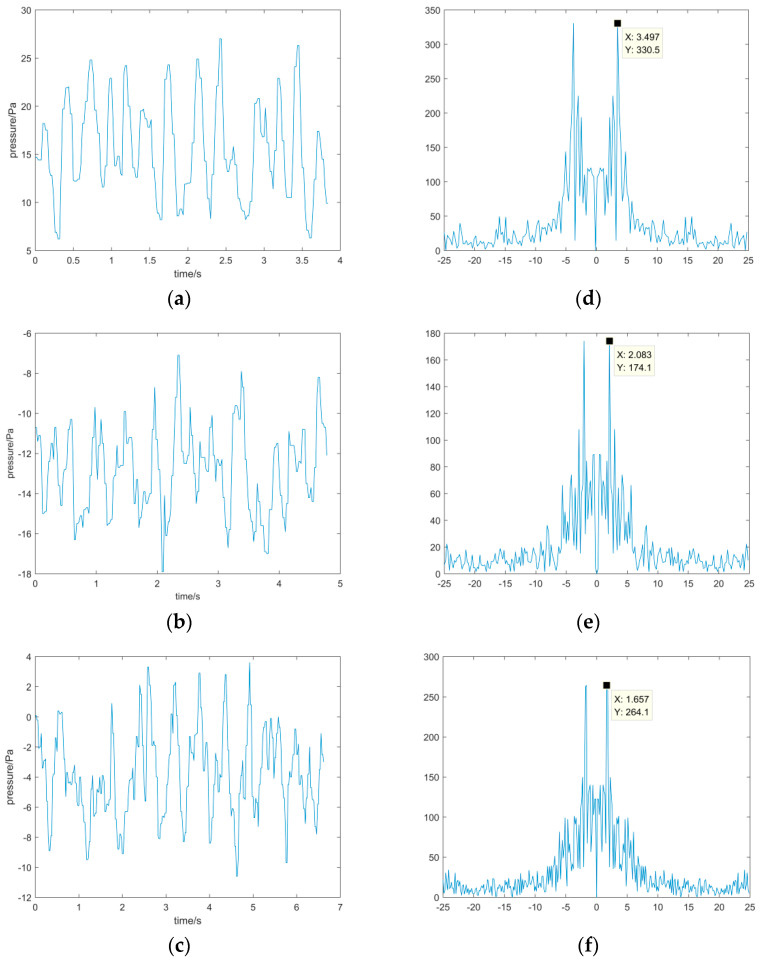
Pressure change in the hole and the frequency spectrum curve of cylinders with different diameters. (**a**) Variation of pressure around a 3 cm cylinder with time. (**b**) Variation of pressure around a 5 cm cylinder with time. (**c**) Variation of pressure around a 9 cm cylinder with time. (**d**) Analysis of pressure spectrum around 3 cm cylinder. (**e**) Analysis of pressure spectrum around 5 cm cylinder. (**f**) Analysis of pressure spectrum around 9 cm cylinder.

When the velocity of the flow field was 10 m/s, three different cylindrical wakes were measured at fixed points. The data of three points A, B, and C shown in Figure 9 were selected for analysis, and the pressure change curve over time was obtained; this is shown in Figure 11. Figure 11a–c show the resulting curves of the pressure measured with the hemispherical seven-hole probe in the flow field of cylinders with different diameters over time. It can be clearly observed that the pressure undergoes periodic changes, which suggests that there are periodic vortices. There are also irregular data spikes that are caused by probe jitters or sensor measurement errors. These errors can be eliminated by spectrum analysis, and the spectrum diagrams are shown in Figure 11d–f. The frequency corresponding to the maximum amplitude point in these spectrum diagrams is the frequency of the periodic change in the hole pressure, i.e., the frequency change of the vortex. The frequency change of the 3, 5, and 9 cm cylindrical vortex were 3.497, 2.083, and 1.658 Hz, respectively. It is clear that as the diameter of the cylinder increases, the frequency change of the vortex gradually decreases.

## 5. Conclusions

To use the multi-hole probe to measure the vortex, a three-coordinate multi-directional rotatable calibration and measurement system was designed and built. A three-coordinate multi-directional rotatable mobile bracket was used to fix the probe and control its movement in three-dimensions according to yaw and pitch rotation, with rotation of up to 360° around the axis. The host computer software sent commands, controlled the motor movement and data acquisition, and completed the probe calibration and measurement in any direction and at any position. Through the measurement of the flow field around different sized cylinders, it was verified that this three-coordinate, multi-directional rotatable probe calibration measurement system can better complete the calibration and measurement tasks of the multi-hole probe. After mining the multi-hole probe data, vortices within the flow field that periodically dropped around the cylinder were detected. When the wind speed was 10 m/s, the shedding frequency of the 3, 5, and 9 cm cylindrical vortex were 3.497, 2.083, and 1.658 Hz, respectively. This verifies that the measurement system is capable of simultaneous measurements of velocity and vortex.

Due to the limited size of the wind tunnel test cabin used for the experiment, only a specific part of the test area was selected for the flow field test. Any point in the flow field can be measured if the flow field is tested in an open test environment. In addition, the pressure sensor can be directly welded to the end of the probe in future work, which can greatly shorten the frequency response time and meet the needs of rapidly changing flow field measurements.

## Figures and Tables

**Table 1:** Wind tunnel technical specifications.

Technical Indicators	Value
Diameter of the experimental section	600 mm
Length of the experimental section	1000 mm
Shrinkage ratio	1:6
Wind speed range	0.2–40 m/s
Uniformity of the flow field	≤1%
Turbulence	<0.5%
Wind tunnel size	10,023 × 1970 × 2500 mm

**Table 2:** Test results and errors.

*U* (m/s)	*u* (m/s)	*v* (m/s)	*w* (m/s)	Δ*U*	Δ*θ* (deg)	Δ*ϕ* (deg)
10.1223	9.7632	2.3353	1.2986	1.2236%	0.31	0.92
18.7771	17.8729	5.0146	2.8273	6.1144%	2.85	0.59
29.2892	28.5301	5.6511	3.4578	2.3693%	1.93	1.46
40.0302	38.6928	9.3067	4.3205	0.0754%	0.15	5.10

## Data Availability

The datasets generated and analysed during the current study are not publicly available due [Project confidentiality] but are available from the corresponding author on reasonable request.
